# Effects of an Interdisciplinary Approach in the Management of Temporomandibular Disorders: A Scoping Review

**DOI:** 10.3390/ijerph20042777

**Published:** 2023-02-04

**Authors:** Nicolò Brighenti, Andrea Battaglino, Pierluigi Sinatti, Vanesa Abuín-Porras, Eleuterio A. Sánchez Romero, Paolo Pedersini, Jorge H. Villafañe

**Affiliations:** 1 Scuola di Medicina, Dipartimento di Scienze della Salute, Università degli Studi del Piemonte Orientale, 28100 Novara, Italy; 2 IRCCS Fondazione don Carlo Gnocchi, 20148 Milan, Italy; 3 Physiotherapy Department, Faculty of Sport Sciences, Universidad Europea de Madrid, 28670 Villaviciosa de Odón, Spain; 4 Musculoskeletal Pain and Motor Control Research Group, Faculty of Sport Sciences, Universidad Europea de Madrid, 28670 Villaviciosa de Odón, Spain; 5 Physiotherapy Department, Faculty of Health Sciences, Universidad Europea de Canarias, 38300 La Orotava, Spain; 6 Musculoskeletal Pain and Motor Control Research Group, Faculty of Health Sciences, Universidad Europea de Canarias, 38300 Tenerife, Spain; 7 Physiotherapy and Orofacial Pain Working Group, Sociedad Española de Disfunción Craneomandibular y Dolor Orofacial (SEDCYDO), 28009 Madrid, Spain

**Keywords:** temporomandibular disorders, physical therapy modalities, dentistry, Interdisciplinary Health Team, musculoskeletal manipulations, exercise therapy, occlusal splint, treatment efficacy

## Abstract

Temporomandibular disorders (TMD) is an umbrella term that encompasses many musculoskeletal problems that include the masticatory muscles, the temporomandibular joint, and other associated structures. TMD can be divided into two large groups: those that affect the musculature and those that affect the joint. The treatment of TMD requires the combined skills of physiotherapists and dentists, as well as sometimes psychologists and other medical specialists. This study aims to examine the effectiveness of the interdisciplinary approach using physiotherapy and dental techniques on pain in patients with temporomandibular disorders (TMDs). This is a Scoping Review of studies investigating the effects of combined therapy on patients with TMD. PRISMA guidelines were followed during this review’s design, search, and reporting stages. The search was carried out in the MEDLINE, CINHAL, and EMBASE databases. A total of 1031 studies were detected and analyzed by performing the proposed searches in the detailed databases. After removing duplicates and analyzing the titles and abstracts of the remaining articles, six studies were ultimately selected for this review. All the included studies showed a positive effect on pain decreasing after a combined intervention. The interdisciplinary approach characterized by the combination of manual therapy and splint or electrotherapy can positively influence the perceived symptoms; positively decrease pain; and reduce disability, occlusal impairments, and perception of change.

## 1. Introduction

Temporomandibular disorders (TMDs) occur due to excessive and/or prolonged joint overload, influenced by biomechanical factors that may lead to excessive or unbalanced joint loading as well as reduced joint adaptability [1]. Injuries can produce pathological changes in the tissue and mechanical properties of the articular disc, loss of cartilage integrity, pain caused by inflammatory mediators, displacement of the articular disc, alteration and loss of synergy of the condyle–disc–eminence complex, and, finally, can generate greater resistance and functional overload on the TMD [2]. A recent line of research also points to a systemic contribution to the development of joint disorders [3]. TMD is an umbrella term that encompasses many musculoskeletal problems that include the masticatory muscles, the temporomandibular joint, and other associated structures. TMD can be divided into two large groups: those that affect the musculature and those that affect the joint.

The treatment of TMD requires the combined skills of physiotherapists and dentists, as well as sometimes rheumatologists and other medical specialists [4]. In certain cases, a psychologist and additional pharmacotherapy may be necessary. Accurate assessment of TMD is critical to define the primary driver of pain by evaluating function and morphology, not overlooking that, in many cases, orofacial and craniofacial pain depends on muscle issues [5,6].

The effectiveness of TMD treatments is controversial, although it is recognized that multidisciplinary treatment with education and counseling, exercise therapy, manual and invasive physiotherapy, and occlusal splint therapy, in addition to pharmacotherapy in moderate to severe pain, is effective in many patients [7,8,9].

As with other conditions with chronic pain, such as low back pain, in TMD, the psychological and physical factors that influence the disability of patients are of great importance and must be considered when treating them [10]. Manual therapy in the cervical region, for example, demonstrates effectiveness to produce local and segmental hypoalgesic effects if the level of catastrophizing is low or medium [11]. However, if the levels of catastrophizing are high, they may result in a poor outcome after the intervention.

While it is true that some physiotherapists and dentists may claim that an altered occlusion may affect the spine, scientifically, there is no evidence of the beneficial effects of orthodontic treatment on spinal deformity [12]. Furthermore, postural work through exercise likely has a direct impact on decreasing pain that does not correlate with improved posture [13,14].

Since in the treatment of this region, physiotherapists and dentists collaborate, the purpose of this study is to examine the current evidence on the effectiveness of the interdisciplinary approach using physiotherapy and dental techniques on pain in patients with TMD.

## 2. Materials and Methods

This is a Scoping Review of studies investigating the effects of combined therapy on patients with TMD. PRISMA guidelines were followed during this review’s design, search, and reporting stages.

### 2.1. Search Strategy

The literature search was conducted independently by two authors (N.B. and A.B.) on MEDLINE, EMBASE, and CINAHL, for articles published before 1 September 2022, using the keywords “temporomandibular joint disorders”, “physiotherapy”, “dentistry”, “interdisciplinary approach”, “manual therapy”, “exercise therapy”, and “occlusal splint”, combining with Boolean operators and MeSH terms and completing the searching operation with manual research by a search methodology expert. The search strategy was not language-restricted, and only human research was considered, including randomized controlled trials (RCTs) and observational studies (cohort studies and case-control studies).

### 2.2. Review Inclusion Criteria

The selected studies’ participants had to be male or female adults with a diagnosis of TMD based on the homonymous diagnostic criteria (DC/TMD) [2] or any clinical diagnosis based on TMD signs and symptoms (myalgia, myofascial pain with referral, and arthralgia).The interdisciplinary physiotherapy and dentistry approach was the analyzed intervention compared with usual care. The selected outcomes were pain and functionality.

### 2.3. Selection Criteria and Data Extraction

After the independent titles and abstracts screening of the identified studies by two authors (N.B. and A.B.), full texts of the potentially relevant articles were retrieved. All disagreements between the reviewers were settled with another author (J.H.V.). The manual search of relevant studies’ references was applied to retrieve additional articles. Exclusion criteria based on study design were editorials, comments, case reports/series, letters to the editor, reviews, and meta-analyses. The studies that included subjects under 18 years of age and subjects with a history of TMD surgery or systemic pathologies were excluded.

### 2.4. Methodological Quality and Risk of Bias Assessment

RCT methodological quality was evaluated using the PEDro scale. The PEDro scale is an 11-item scale designed to rate randomized clinical trials (RCTs) methodological quality. Each item that is satisfied on the scale contributes one point to the total possible score of 10 points [15]. The methodological index for nonrandomized studies (MINORS) has been used to assess methodological quality and risk of bias of observational studies [16]. The tool comprises 16 and 24 items for nonrandomized studies and comparative studies, respectively, and each item is scored from 0 to 2 [16]. The Cochrane Risk-of-Bias for Randomized Trials (RoB 2) and the Risk Of Bias In Non-randomized Studies - of Interventions (ROBINS-I) tools were used to assess the risk of bias in the RCTs and non-RCT included in the present study. RoB 2 evaluates a set of bias domains, focusing on different aspects of trial design, conduct, and reporting. ROBINS-I included specification of the target trial and effect of interest, use of signaling questions to inform judgments of risk of bias, and assessments within seven domains of bias. It was also used the NIH Quality Assessment Tool for Observational Cohort and Cross-Sectional Studies, based on 14 items that were scored as "cannot determine, not applicable, or not reported". Finally, the results of the NIH quality assessment tool were obtained as " Good, Fair, or Poor”.

## 3. Results

### 3.1. Study Selection

**Figure 1:**
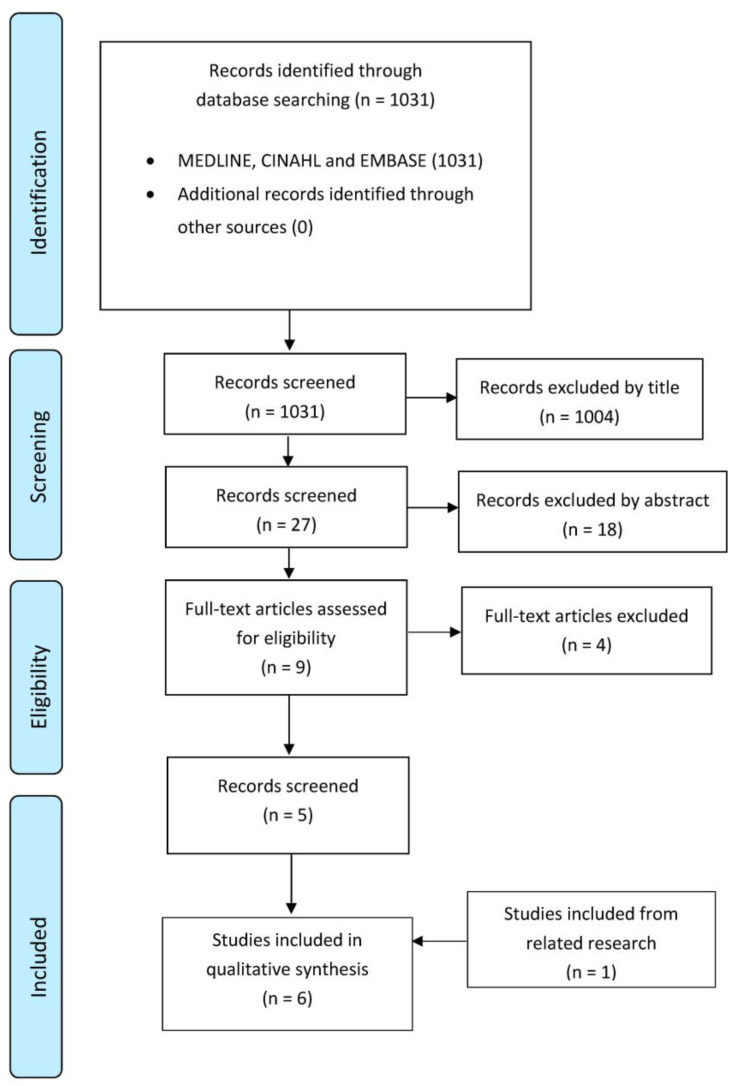
PRISMA Flow diagram.

Initially, 1031 articles were found through the databases search on MEDLINE, CINHAL, and EMBASE. Once duplicates were eliminated, and the titles and abstracts of the remaining papers were examined, nine full-text articles were explored to verify their eligibility for inclusion in this review. Four of these articles were excluded, and lastly, one study was included from related research. Six studies [17,18,19,20,21,22] were finally selected for this study. The selection of these articles through the review procedure is reported in Figure 1 (flow diagram based on PRISMA statement), and the details of the selected studies are gathered in Table 1.

### 3.2. Risk of Bias within and across the Studies

**Figure 2:**
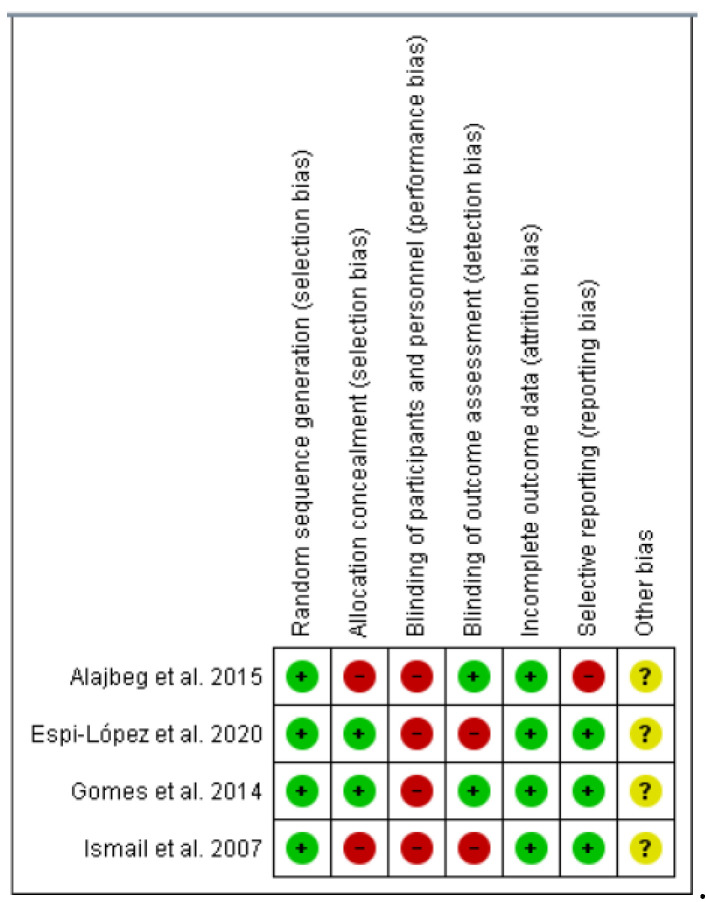
Cochrane risk-of-bias tool for randomized trials (RoB 2) [17,19,21,22].

**Figure 3:**
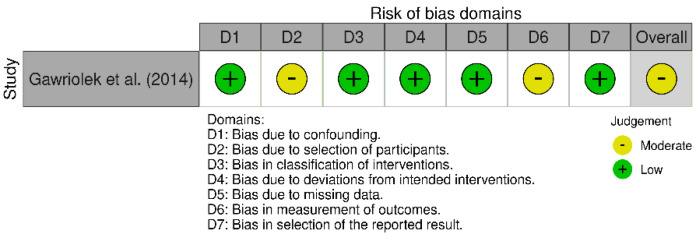
The risk of bias in nonrandomized studies of intervention (ROBINS-I) assessment tool [20].

The risk of bias analysis of the four RCTs included in this review [17,19,21,22] was carried out using the Cochrane risk-of-bias tool for randomized trials (RoB 2). All the studies analyzed results in “high risk” only in blinding items. Results from RoB 2 are summarized in Figure 2. The risk of bias analysis of the Gawriolek et al. study [20] was realized using the Risk Of Bias In Non-randomized Studies - of Interventions (ROBINS-I) and the result was “moderate risk”. Results from ROBINS-I are reported in Figure 3.

### 3.3. Quality Assessment

The PEDro scale was used to assess the quality of the four RCTs included in the review. Three of the articles reviewed [17,19,21] were of good quality (scores 6 and 8), and one study, the one realized by Isamil et al. in 2007 [22], was of fair quality (score 4–5). The MINORS scale was used to evaluate the quality of the article realized by Gawriolek et al. [20] that resulted in “good quality”. The NIH quality assessment tool for cross-sectional and observational cohort studies was used to evaluate the quality of the article realized by Toledo Jr et al. [18] that resulted in “good quality”. The results of the PEDro scale, the MINORS scale, and the NIH quality assessment tool can be found in Table 1.

### 3.4. Studies Report

The study realized by Espí-Lopez et al. [19] was conducted to ascertain whether a combined program of manual therapy (MT) plus traditional splint therapy (ST) improves pain and function in subjects with TMD. Sessions of forty-five minutes of combined MT techniques were realized for 4 weeks, one time a week, and three evaluations were registered: at baseline, post-treatment, and one-month follow-up. Results of VAS revealed that subjects in the EG had significantly improved pain scores at T1 (*p* = 0.001), which was maintained at T2 (*p* = 0.001), with the treatment factor explaining 33.2% (R2) of pain-score improvement with a large effect size (f2: 0.50). About the distinctions between groups, statistical differences were showed at T1 and T2 for VAS, with a large effect (*p* = 0.001, Cohen’s d = 0.8 for both). The results of the Helkimo Index revealed a significant reduction of 4.4 points, between T0 and T1, in the EG (*p* = 0.001). The CG, however, showed no statistical differences inside the group. Regarding pain pressure threshold (PPT) there was a significant improvement in the EG for all the TM muscles, whereas the CG remained at similar values for the entire experimentation time. The group variances were statistically significant for all the analyzed muscles at T1 and T2. About the effect of the intervention over the course of time: in the EG at T1, the algometry of the three muscle groups improved significantly, and the Helkimo and the VAS both reduced significantly. These improvements were maintained at T2. The Patient Global Impression of Change Scale (PGICS) showed that, at T2, the EG perceived a greater improvement after treatment (CG: 4.3, SD = 0.9; EG: 2.4, SD = 1.4), and this difference between groups was statistically significant (mean difference = 1.9, *p* = 0.005). In conclusion, this study showed that MT combined with ST leads to a decrease in pain (3-point decrease), higher PPT (of at least 1.0 kg/cm^2^), improvement of disability caused by pain (4.4-point decrease), and positive perception of change (EG: 50% felt “much improvement”), compared to ST alone.

The aim of the study realized by Gomes et al. [21] was to analyze the effects of massage therapy (MT), conventional occlusal splint therapy (COST), and silicone occlusal splint therapy (SOST) on the intensity of signs and symptoms in subjects with sleep bruxism (SB) and severe TMD and electromyographic activity in the muscles masseter and anterior temporal. Groups were evaluated using electromyographic analysis of the masseter and anterior temporal muscles and the Fonseca Patient History Index. The assessment was realized before and after the intervention. In the intragroup analysis, no statistically significant differences were found between the pre- and post-intervention assessments of the masseter and anterior temporal muscles in the groups. In the Fonseca Patient History Index, differences were found between the pre- and post-intervention evaluations in the MT, COST, and SOST groups, the latter of which exhibited greater improvement in comparison to the other groups. In conclusion, the results of this study showed that the use of SOST and MT had no significant impact on the electromyographic activity of the masseter or anterior temporal muscles, but the combination of interventions led to a decrease in the signs and symptoms of subjects with severe SB and TMD.

The study realized by Toledo et al. [18] analyzed three factors: (1) therapy, which was transcribed physical therapy modalities used; (2) if there were any procedures for home treatment without a professional; and (3) the temporomandibular joint (TMJ) palpation pain rates, measured with a visual analog scale (VAS). Chi-square analysis showed that an interdisciplinary therapy between physiotherapy and dentistry is effective in TMD pain reduction (*p* = 0.014). Statistical analysis of these comparisons, the Chi-square, showed that all physiotherapy modalities contributed to TMD pain reduction. The statistical analysis was realized by the Chi-square test, and results showed that physical therapy procedures performed without the presence of professionals for TMD treatment are very helpful in pain reduction (*p* = 0.002). In conclusion, the study realized by Toledo et al. [18] showed that all physical therapy modalities contributed to TMJ pain reduction. Guidelines for physiotherapy at home proved to be very helpful in reducing pain. The effectiveness of interdisciplinary work in physiotherapy and dentistry treatments for TMD has been adequately verified.

The work produced by Ismail et al. [22] was realized to evaluate the effects of physiotherapy combined with splint therapy in subjects with temporomandibular disorders (TMDs). Before treatment, a subjective pain level was assessed by VAS, and an electronic recording and clinical examination of jaw movements were performed, and a second evaluation was performed after 3 months. The analysis of treatment outcomes realized in this study showed that all analyzed variables improved significantly during the intervention in comparation with the baseline in both groups. Results showed that active jaw opening was significantly higher in group 2 after intervention (*p* < 0.05). In contrast, the were no statistically significant difference between groups for the passive jaw opening. Analysis of subjective pain evaluation in group 1 showed an improvement in total pain intensity, pain intensity during mandibular movement, pain intensity without mandibular movement, and pain intensity after mandibular loading (*p* < 0.05). Similarly, pain intensity in group 2 reduced after intervention (*p* < 0.05). No significant differences were found between the two groups for the subjective parameters. In conclusion, the results of this study showed that physical therapy combined with temporomandibular spin seems to have a positive effect in patients with TMD. 

The study realized by Alajbeg et al. [17] was realized to evaluate the combination of occlusal splint (SS) and physical therapy (PT) for the treatment of anterior disc displacement without reduction. An assessment of VAS was conducted at baseline (T0), and then after 1 month (T1), 3 months (T2), and 6 months (T3). In the treatment outcomes, there were pain-free opening (MCO), maximum assisted opening (MAO), and path of mouth opening. According to VAS, the mean values for the worst pain at baseline for the experimental (SS combined with PT) and control (SS alone) groups were 74/100 and 65.3/100, respectively. The intensity of pain was showed to decrease continuously across time; the difference was significant between experimental (F = 28.964, *p* = 0.0001, effect size = 0.853) and control (F = 8.794, *p* = 0.001, effect size =0.638) groups. Results showed that pain-free opening and maximum assisted opening improved notably in the course of time only in group 1 (MCO: F = 20.971, *p* = 0.006; MAO: F = 24.014, *p* = 0.004). Using an occlusal splint alone did not lead to statistically significant improvements in the amount of mouth opening (*p* > 0.05). In conclusion, this study provided evidence that the combination of SS with PT was more effective than the use of SS alone in decreasing deviations and improving the range of mouth opening in a treatment period of 6 months. Moreover, both interventions reduced pain in subjects with anterior disc displacement.

In the study realized by Gawriołek et al. [20], the jaw-tracking records (K7, Myotronics-Noromed Inc., Washington, DC, USA) were performed, including the measurements of opening, lateral, and protrusive ROM, and the maximal and average velocity of opening and closing. The treatment involved daily stretching movements combined with nocturnally applying a nonoccluding sublingual relaxation splint. After the intervention, both groups presented patients who reported significantly reduced pain. After 6 months of therapy, the success rate of no pain was 40% and the functional impairment of the stomatognathic system decreased, with an average successful outcome of 86% (*p* < 0.05). Results showed a significant decrease in the occurrence of muscle pain during free movement and TMJ pain on palpation with an average successful outcome of 81%. The change in joint clicking was nonsignificant. Results showed that the opening ROM significantly increased by 8 mm (19%). The accompanying deviations and the constituent values of this movement also increased. The protrusion movement showed no statistical difference from the healthy group, but the lateral movement range improved on both sides by an average of 2.1 mm (both sides 36%). In functional evaluations, a satisfactory result was registered by 86% of the participants. In conclusion, the results of this study showed how myorelaxation therapy was effective in the treatment of TMD. After six months of intervention, a significant improvement in opening/closing velocity, opening movement range, and lateral movement range was obtained.

## 4. Discussion

The main objective of this scoping review was to examine the current evidence on the effectiveness of the interdisciplinary approach using physiotherapy and dental techniques on pain in patients with TMDs. In terms of the effectiveness of the multidisciplinary approach, three of the RCTs [17,19,21] included in the methodological quality analysis showed “good quality” and “some concerns” but not “high risk” in terms of risk of bias, Only one RCT [22] showed methodological “fair quality” and “some concerns” of risk of bias. In addition, an observational study included in quality assessment [18] showed “good quality”, and another showed “good quality” and a moderate risk of bias [20].

For example, one RCT of “a fair quality” [22] found that, in a group of 13 patients with TMD, physiotherapy in combination with Michigan occlusal splint therapy had a positive effect in improving mandibular movement capacity and decreasing pain, compared to the only Michigan occlusal splint therapy group. As a physiotherapy protocol, passive traction and translation movements in all restricted directions were used, in addition to the exercise of the levator mandibularis muscles. However, the lack of a larger sample size (although they indeed met the appropriate sample size and dropout calculation requirements) and adequate description of the physical therapy protocol means that the results may be biased concerning the attribution of manual therapy as responsible for the observed effects. In addition, it remains a pre–post study, although all patients were treated and measured between the first, fourth, eighth, and twelfth week, there were no midterm or long-term measurements after the end of treatment. In a recent systematic review performed to investigate the medium- and long-term efficacy of manual therapy as a management of TMD, alone or in combination with therapeutic exercise, Herrera-Valencia et al. [23] described a significant reduction in pain and mouth opening relative to baseline values after treatment with manual therapy. These authors suggested manual therapy for its medium-term effects (even though the impact seemed to decrease with time) in combination with therapeutic exercise, as this way its effects are preserved in the long term. In another recent systematic review, Zhang et al. [24] found no high-quality evidence to discriminate clinical efficacy from occlusal splinting to exercise therapy for patients with TMD pain. Although the techniques manual therapy may use are not determinant, therapeutic success can be attributed to the correct diagnosis and adequate combination of multidisciplinary treatment [6]. A recent RCT by Urbański et al. [25] evaluated the efficacy of two different physiotherapy techniques in 70 patients affected by TMD with a dominant muscle component who were divided into two groups: patients in group I underwent postisometric relaxation treatment, and patients in group II were treated with myofascial release treatment. After ten treatments, no significant differences were observed between the groups in terms of pain intensity, and in both groups, there was a significant decrease in the electrical activity of the masticatory muscles examined.

In the same way, one good quality observational study [18] evaluated the interdisciplinary work between dentistry and physiotherapy in 300 patients affected by TMD, highlighting the importance of implementing an interdisciplinary treatment program, in which physiotherapy helps to relieve pain and dentistry treats disorders related to the stomatognathic system. According to these observations, Al-Moraissi et al. [26] suggest modifying the appropriate practice of exhausting conservative treatment options to the detriment of minimally invasive procedures, such as arthrocentesis, as soon as patients do not show a clear benefit from initial conservative treatment. In their network meta-analysis of randomized clinical trials, these authors highlight that there is evidence (albeit at a very low to moderate level of quality) that the use of hyaluronic acid, corticosteroid, or platelet-enriched plasma infiltrations is significantly more efficacious than conservative treatments in both reducing pain and improving maximum mouth opening in the short (≤5 months) and medium term (6 months–4 years). Furthermore, those authors note that noninvasive procedures delivered significant inferior-quality results in terms of pain and maximal oral opening. However, in our opinion, this study is highly biased: the only physiotherapy techniques analyzed were manual therapy and laser therapy, ignoring techniques such as dry needling, which reaches an effectiveness level of evidence 1a in TMD of myogenic origin [27]. In addition, these authors analyze exercise within conservative treatments, which include flat stabilization splinting or anterior-repositioning splinting, home muscle exercise, and self-care, which leads to a very generic and superficial analysis of exercise.

Furthermore, the type of exercise may be very important for achieving successful outcomes, such as therapeutic exercise based on scientific evidence [28]. Bouchard et al. [28] and Vos et al. [29] reported a lack of evidence to support arthrocentesis as a better therapeutic intervention than nonsurgical interventions. Furthermore, they underlined the prominent role of conservative treatments such as physical therapy and oral/topical and injected pharmacotherapies and suggested their superiority over surgery, since they are less invasive and usually produce satisfying clinical results in mild-moderate TMD [30,31,32,33,34,35,36,37,38,39,40,41,42,43,44].

In the quasiexperimental study by Gawriolek et al. [20], the examination of the treated TMD group revealed that 19 patients had muscle involvement and 31 patients were diagnosed with TMJ disc displacement with reduction. Interestingly, the follow-up period conducted by the researchers, from 4 weeks post-treatment to 3 and 6 months later, determined that the sublingual relaxation splint coupled with everyday active stretching exercises were shown to be efficacious in the group of TMD patients who suffered from muscle impairment and disc displacement with a reduction in the improvement of mandibular range of motion and self-perceived pain. However, according to a recent systematic review by Armijo-Olivo et al., there is no clear evidence of the superiority of exercises over other conservative treatments for TMDs [31].

In terms of the effectiveness of the interdisciplinary approach, two of the three RCTs [17,19,21] were pilot studies, consisting of only 12 [17] and 16 [19] patients in each study, respectively. In the first study [17], the groups were six patients who were assigned to manual therapy plus splinting (experimental group) or splint therapy alone (control treatment), and eight patients who were assigned to similar groups in the other study [19]. Although both studies found improvements in pain and motion in patients using the two types of treatment, the small sample size of the studies mean that the conclusions of the studies mut be taken with some distance. However, Espí-López et al. [19] used a more extensive manual therapy protocol comprising a combination of ten techniques applied to the cervical, suboccipital, and temporomandibular areas, while the study by Alajbeg et al. [17] focused on the muscles and joints of the mandibular region. We know that better results are obtained to treat the pain and dysfunction of patients affected by TMD if, in addition to evaluating and treating the most affected region of the jaw, we perform treatments at a distance, such as in the region most anatomically and neurophysiologically interconnected with it, which is the neck [45]. Furthermore, we know that patients with TMD more frequently exhibited cervical spine pain than people without craniomandibular pain, regardless of the classification model utilized, since the craniomandibular system and the cervical spine are generally considered to be a functional entity [46].

Finally, the study by Gomes et al. [21] did have an adequate sample of patients to answer their hypothesis, which was similar to that proposed by the two previous groups of authors, reaching similar conclusions: improved range of motion and decreased pain in patients affected by TMD if a splint + manual therapy are combined. 

However, in only one [17] of the three RCTs included in this scoping review was the positive presence of a psychiatric record considered as an exclusion criterion for participation in the study, and in none were psychological variables assessed, either as inclusion or exclusion criteria, but as part of the variables to be analyzed. The authors of the present review consider this to be an error since in the case of TMD, the patient’s psychological state may have a significant influence on the onset and development of complications of this disorder, as demonstrated by the increase in stress, anxiety, and depression in patients with TMD [47,48].

It should be noted that, although sleep in patients was not analyzed in the included studies, a recent systematic review found that there is insufficient evidence to suggest that patients with temporomandibular joint osteoarthritis are associated with increased sleep disorders or poorer sleep quality [49].

It is recognized that the sample of studies included was low. Even so, in return, it was decided to analyze the studies that used physical therapy and dental techniques together rather than separately to analyze the pure relationship between this type of interdisciplinary approach. A meta-analysis could not be performed due to the considerable heterogeneity of the studies included, which must be viewed as a limitation of the study. However, the scoping review performed widely responds to the objectives established.

## 5. Conclusions

The interdisciplinary approach characterized by the combination of manual therapy and splint or electrotherapy can influence the perceived symptoms positively, showing a positive effect on pain decreasing, reduction in disability, occlusal impairments, and perception of change. Future interdisciplinary research in physiotherapy and dentistry should include large sample sizes of experimental studies and further investigation of the development of physiotherapy and dentistry techniques used in clinical practice to treat patients with TMD.

## Figures and Tables

**Table 1:** Characteristics of included studies.

Authors, Years	Aim of the Study	Study Design, Participants, Methods	Outcomes + Follow-Up	Results	Conclusions	Quality Score
Ismail et al. [22] (2007)	To evaluate the efficacy of physical therapy, in addition to splint therapy, on treatment outcomes in patients with TMD with respect to objective and subjective parameters.	Study design: RCT N: 26 patients diagnosed with TMD. - 13 patients treated with occlusal splint only (group I). - 13 patients treated with occlusal splinting and physiotherapy (group II).	Jaw mobility Active and passive maximum jaw opening Pain Visual Analogue Scale (VAS) - Total pain intensity - Pain intensity during, without, and after mandibular movement Follow-up: 1, 4, 8, and 12 weeks	Compared with the baseline, in both groups, mandibular movement capacity increased significantly after treatment, whereas subjective pain decreased significantly (*p* < 0.05). After therapy, the difference in active jaw opening between groups was significant (*p* < 0.05).	Physiotherapy, combined with occlusal splint therapy, appears to positively affect the treatment outcome of patients with TMD.	PEDro 5/10
Gomes et al. [21] (2014)	To evaluate the effects of manual therapy and occlusal splints on the electromyographic activity and signs and symptoms of patients with severe TMD.	Study design: RCT N: 60 participants diagnosed with TMD were randomly distributed into 4 groups. (1) massage group. (2) conventional occlusal splint group. (3) massage group + conventional occlusal splint group. (4) silicone occlusal splint group.	Electromyographic activity Surface EMG (right and left masseter and anterior temporal muscles) Follow-up: None, only pre- and post-treatment evaluations	All groups had a statistically significant improvement compared to before the intervention. Comparing the groups, only the group that combined manual therapy with occlusal splinting had a statistically significant improvement (*p* < 0.05).	The combination of massage and conventional occlusal splinting reduced the intensity of signs and symptoms among individuals with severe TMD.	PEDro 7/10
Alajbeg et al. [17] (2015)	The hypothesis tested was that the simultaneous use of an occlusal splint and physical therapy is an effective treatment of anterior disc displacement without reduction (TMD).	Study design: RCT N: 12 participants with anterior disc displacement without reduction (TMD) were randomly assigned into 2 groups: - 6 received an occlusal splint (group I). - 6 received both physiotherapy and occlusal splint (group II).	Pain Visual Analogue Scale (VAS) for spontaneous pain Pain-free opening and path of mouth opening Maximum comfortable opening (MCO) Maximum assisted opening (MAO) Opening pattern Follow-up: 1 month, 3 months, and 6 months	Regarding VAS scores, group II had a greater improvement (*p* = 0.0001) than group I (*p* = 0.001). Jaw opening with and without pain improved significantly only in group II (maximum opening without pain *p* = 0.006, maximum opening with pain *p* = 0.004). The use of the occlusal splint alone did not lead to statistically significant changes in the range of mouth opening (*p* > 0.05).	The simultaneous use of an occlusal splint and physiotherapy improved the range of mouth opening more effectively than the occlusal splint used alone. Both treatment options were effective in reducing pain.	PEDro 6/10
Espí-López et al. [19] (2020)	To ascertain whether a combined program of manual therapy techniques, including intraoral treatment, plus traditional splint therapy improves pain and clinical dysfunction in subjects with TMD.	Study design: RCT N: 16 participants with TMD were assigned to either the manual therapy plus splint—experimental group (EG, *n* = 8) or the splint therapy alone—control group (CG, *n* = 8).	Pain Visual Analogue Scale (VAS) Pain pressure threshold (PPT) Minimal pressure which induces pain (pressure algometry) Dysfunction Index of TMD Helkimo Index Change perception Patient Global Impression of Change Scale (PGICS) Follow-up: 1 month	EG showed a significant reduction in pain, higher pain pressure threshold, significant improvement of dysfunction, and significantly positive perception of change after treatment (*p* < 0.05 all). The between-group differences were statistically significant.	Manual therapy plus splint therapy showed a reduction in perceived pain (3-point decrease), higher pain pressure threshold (of at least 1.0 kg/cm^2^), and improvement of disability caused by pain (4.4-point decrease), compared to splint therapy alone.	PEDro 6/10
Toledo Jr et al. [18] (2012)	This study aims to evaluate the effectiveness of interdisciplinary work between dentistry and physiotherapy in determining the treatment plan for patients with TMD.	Study design: A retrospective cohort study N: 300 patient records from the ATM service file. Three factors were analyzed: (1) The physiotherapy techniques used. (2) Guidelines or procedures for home exercises. (3) the temporomandibular joint (TMJ) palpation pain rates (VAS).	Temporomandibular joint pain rates Visual Analogue Scale (VAS)	The initial analysis of pain on palpation showed that 151 patients (50.3%) had a high level of pain (grade III), 92 (30.7%) had a moderate level of pain (grade II), 39 (13%) had a low level of pain (grade I), and 18 (6%) had no pain. Considering the final pain on palpation, 13 patients (4.3%) had grade III, 22 (7.3%) grade II, 63 (21%) grade I, and 202 (67.3%) were without pain.	It is essential to have an interdisciplinary treatment plan, where physical therapy helps pain relief and dentistry treat the disorders related to the stomatognathic system.	NIH “Good” quality.
Gawriolek et al. [20] (2014)	This study aimed to analyze the effectiveness of myorelaxation therapy (nocturnal sublingual splint + stretching exercises) in TMD.	Study design: nonrandomized controlled trial N: 78 participants. - The first group consisted of 32 patients suffering from TMD who served as the treated group. - The second group consisted of 46 volunteers as a healthy group.	Jaw-tracking examination Computerized mandibular scanner (CMS) Functional examination Active mandibular movements Follow-up: After 4 weeks with no intervention, then 3 weeks, 3 months, and 6 months after treatment	After the treatment, mandibular opening range increased by 8 mm (19%, *p* < 0.05), lateral movement by 2.1 mm (36%, *p* < 0.05), while protrusive movement decreased by 0.5 mm (*p* > 0.05). These results were supported by the decrease in reported impairment and clinical pain occurrence (*p* < 0.05).	Myorelaxation therapy was effective in the treatment of the patient group. A significant improvement in opening range, lateral movement, and referred pain was achieved after 6 months of treatment.	MINORS 17/24

## Data Availability

The data presented in this study are available on request from the corresponding authors.
